# A comprehensive insight into the molecular and cellular mechanisms of the effects of Propolis on preserving renal function: a systematic review

**DOI:** 10.1186/s12986-021-00639-z

**Published:** 2022-01-20

**Authors:** Paniz Anvarifard, Maryam Anbari, Alireza Ostadrahimi, Mohammadreza Ardalan, Zohreh Ghoreishi

**Affiliations:** 1 Student Research Committee, School of Nutrition and Food Sciences, Tabriz University of Medical Sciences, Tabriz, Iran; 2 Nutrition Research Center, Tabriz University of Medical Sciences, Tabriz, Iran; 3 Department of Clinical Nutrition, School of Nutrition and Food Sciences, Tabriz University of Medical Sciences, Attar-Neishaburi St., Golgasht Alley, Azadi Blvd., Tabriz, Iran; 4 Kidney Research Center, Tabriz University of Medical Sciences, Tabriz, Iran

**Keywords:** Propolis, Acute kidney injury, AKI, Chronic kidney disease, CKD, Renal function, Kidney disease, Systematic review

## Abstract

**Background:**

The present systematic review is conducted, focusing on the existing evidence of Propolis's effects due to its various health benefits, mainly antioxidant and anti-inflammatory properties on preserving renal function.

**Methods:**

A systematic search of PubMed, Scopus, Embase, ProQuest, and Google Scholar was undertaken for relevant papers published from the start until January 2021.

**Results:**

This review revealed that Propolis affects fasting blood sugar (FBS), postprandial blood glucose, advanced glycation end products (AGEs) concentrations, malondialdehyde (MDA) levels, urinary concentrations of reactive oxygen metabolites (Tbars), total oxidant status (TOS), oxidative stress index (OSI), and 8-hydroxy-2′-deoxyguanosine (8-OHdG) formation favorably. The findings on hemoglobin A1C (HbA1C), insulin, homeostasis model assessment of insulin resistance (HOMA-IR), β-cell function (HOMA-β), quantitative insulin sensitivity check index (QUICKI), and lipid profile were controversial. Moreover, a significant reduction in renal nuclear factor kappa B (NF-κB), serum immunoglobulins, renal ED-1^+^ cells, and urinary monocyte chemoattractant protein-1 (MCP-1) following Propolis supplementation has been reported, while the results on interleukin-6 (IL-6), tumor necrosis factor α (TNF-α), nitric oxide (NO), nitric oxide synthetase (NOS), and high sensitivity C-reactive protein (hs-CRP) were controversial. Furthermore, included studies showed its anti- proteinuria and kidney restoring effects.

**Conclusion:**

In this review, both human and animal studies provide us evidences that Propolis could potentially improve the glycemic status, oxidative stress, renal tissue damage, and renal function. Further studies are needed to determine the underlying mechanisms.

**Supplementary Information:**

The online version contains supplementary material available at 10.1186/s12986-021-00639-z.

## Introduction

Kidney disease is a serious global health challenge with a growing prevalence [1, 2] and chronologically is divided into two main categories, acute kidney injury (AKI) and chronic kidney disease (CKD) [1]. AKI affected 10–20% of hospitalized adults and up to 60% of critically ill patients worldwide in 2015 [2, 3]. The current diagnostic approach of AKI, is based on an acute decrease of glomerular filtration rate (GFR), reflected by an acute rise in serum creatinine (SCr) levels and/or a decline in urine output over a given time interval. The leading causes of AKI are hospital-acquired (renal ischemia, sepsis, and nephrotoxic drugs or herbals) and community-acquired (such as infections, diarrhea, and dehydration) diseases [3]. On the other hand, the global prevalence of CKD in 2015 was 11–13%, with the majority of stage 3, in non-hospitalized adults [2, 4]. According to the current guidelines, CKD is defined by gradual and permanent decreased renal function (GFR) and/or presence of kidney damage (based on imaging or proteinuria) for more than 3 months, irrespective of the underlying cause [2, 4, 5]. The major causes of CKD are diabetes mellitus (DM), hypertension (HTN), infections, genetic diseases (such as polycystic kidney disease), and autoimmune diseases (such as immunoglobulin A glomerulonephritis and lupus) [1, 5]. Diabetic kidney disease (DKD) is a kind of CKD that occurs in diabetic patients, clinically defined as diabetic nephropathy (DN) (the presence of albuminuria, impaired GFR (< 60 mL/min/1.73m^2^), or both) encompasses with atheroembolic disease, ischemic nephropathy, and interstitial fibrosis [6–8].

Kidney disease, as a silent killer, leads to various health complications, including frailty, hospitalization, cognitive dysfunction, reduced quality of life, end-stage renal disease (ESRD), cardiovascular disease (CVD) as the leading cause of death in the world, and premature mortality [2, 4–6, 9, 10]. Therefore, the prevention and early detection and treatment of kidney disease can be a practical approach for the global decline in ESRD, CVD, and total mortality [2, 5]. Based on the present evidence, it seems that hyperglycemia, dyslipidemia, oxidative stress (OS), and inflammation are four critical parameters for AKI and CKD pathogenesis; indeed, they are considered as causes and/or consequences of kidney disease [1, 11, 12].

Although various medications are available in the market to control and reduce kidney disease complications, new remedies with more therapeutic benefits and less toxicity still are needed [13]. Nowadays, natural products have been highly considered for their role in alleviating OS and inflammation, which might prevent kidney disease progression, as well as complications' reduction also [10, 13]. Propolis, due to its polyphenolic content, multi-targeted effectiveness, and low toxicity is a good candidate [14]. Honeybees make Propolis by mixing their saliva containing specific enzymes and beeswax with exudate collected from plants, and it contains multiple polyphenolic compounds, mostly flavonoids and phenolic acids [14, 15]. Propolis has widely used to treat various diseases due to its antimicrobial, antiseptic, antiulcer, anti-cavity, anti-inflammatory, antioxidant, anticancer, antihypertensive, antiplatelet, and immunomodulatory properties [14, 16–18]. Notably, Propolis may lower the development of neurodegenerative disorders, cancer, diabetes, liver and kidney injuries, immune diseases, and cardiovascular events through its antioxidant properties. It is also supposed that Propolis may attenuate the adverse effects of chemotherapeutic agents [15, 16]. Clinical studies in both mice and humans show that Propolis and its compounds are usually well-tolerated and are non-toxic if used in moderation [14].

Despite the number of studies that investigated the effects of Propolis on metabolic indices such as glycemic status, lipid profile, OS, inflammation, as well as renal function in kidney disease [10, 13, 19–33], there is no comprehensive assessment of the existing evidence based on our review. Therefore, the purpose of this systematic review was to summarize the available data and compare the results of the human and animal studies on the effects of Propolis on metabolic status and renal function.

## Material and methods

### Search strategy

To identify the eligible studies for this systematic review, a search of PubMed, Scopus, Embase, ISI Web of Science, ProQuest, and Google Scholar online databases was conducted from the start up to January 2021, using the key words (“Propolis” [MeSH Terms] OR “Propolis” [Title/Abstract] OR “Bee glue” [Title/Abstract] OR “Bee bread” [Title/Abstract] or “Honeybee” [Title/Abstract] OR “Chrysin” [Title/Abstract]) **AND** (“kidney” [MeSH Terms] OR “kidney” [Title/Abstract] OR “renal” [MeSH Terms] OR “renal” [Title/Abstract] OR “nephropathy” [Title/Abstract] OR “glomerular filtration rate” [Title/Abstract] OR “GFR” [Title/Abstract] OR “Albuminuria” [MeSH Terms] OR “Albuminuria” [Title/Abstract] or “Microalbuminuria” [Title/Abstract] OR “Macroalbuminuria” [Title/Abstract] OR “Proteinuria” [Title/Abstract] OR “Creatinine” [Title/Abstract] OR “dialysis” [MeSH Terms] OR “dialysis” [Title/Abstract] OR “Haemodialysis” [Title/Abstract] OR “Catheter-related bloodstream infections” [Title/Abstract] OR “Central venous catheters” [Title/Abstract]). The search was restricted to clinical trials and animal studies published only in the English language. Guideline of the Preferred Reporting Items for Systematic Reviews and Meta-Analyses (PRISMA) was used for designing and reporting this systematic review (see Additional file 1).

### Inclusion and exclusion criteria

After removing the repeated articles, the titles and abstracts of all imported studies were screened by two independent researchers (P. A. and M. A.). Studies were eligible for inclusion if they meet the following criteria: (1) clinical trial or animal study, (2) publishing in English language, and (3) evaluating Propolis administration effect on kidney disease (only AKI or CKD). Studies were excluded if they 1) were reviews, conference papers, observational studies, or abstracts only, (2) used in-vitro models, (3) investigated other kinds of kidney disease (such as sepsis-related kidney disease or medication-induced kidney toxicity), (4) used some specific compound of Propolis (such as chrysin), and 5) were published in a non-English language.

### Selection, extraction, and assessment of study quality

Two investigators (P. A. and M. A.) screened titles and abstracts of all imported studies to identify articles requiring full-text review using a standardized checklist of the research question and inclusion and exclusion criteria. Any disagreements between the researchers were resolved through consensus. Then, the quality of the included articles was checked by the third investigator (Z. G.). Finally, the following variables were extracted from included studies into a standardized template: first author's name, publication date, study location, type of study, cause of kidney disease, samples characteristics (gender, weight, age, sample size, and groups' allocation), study design, daily dose, duration, and route of Propolis administration, and the main results.

## Results

### Selected articles

**Fig. 1:**
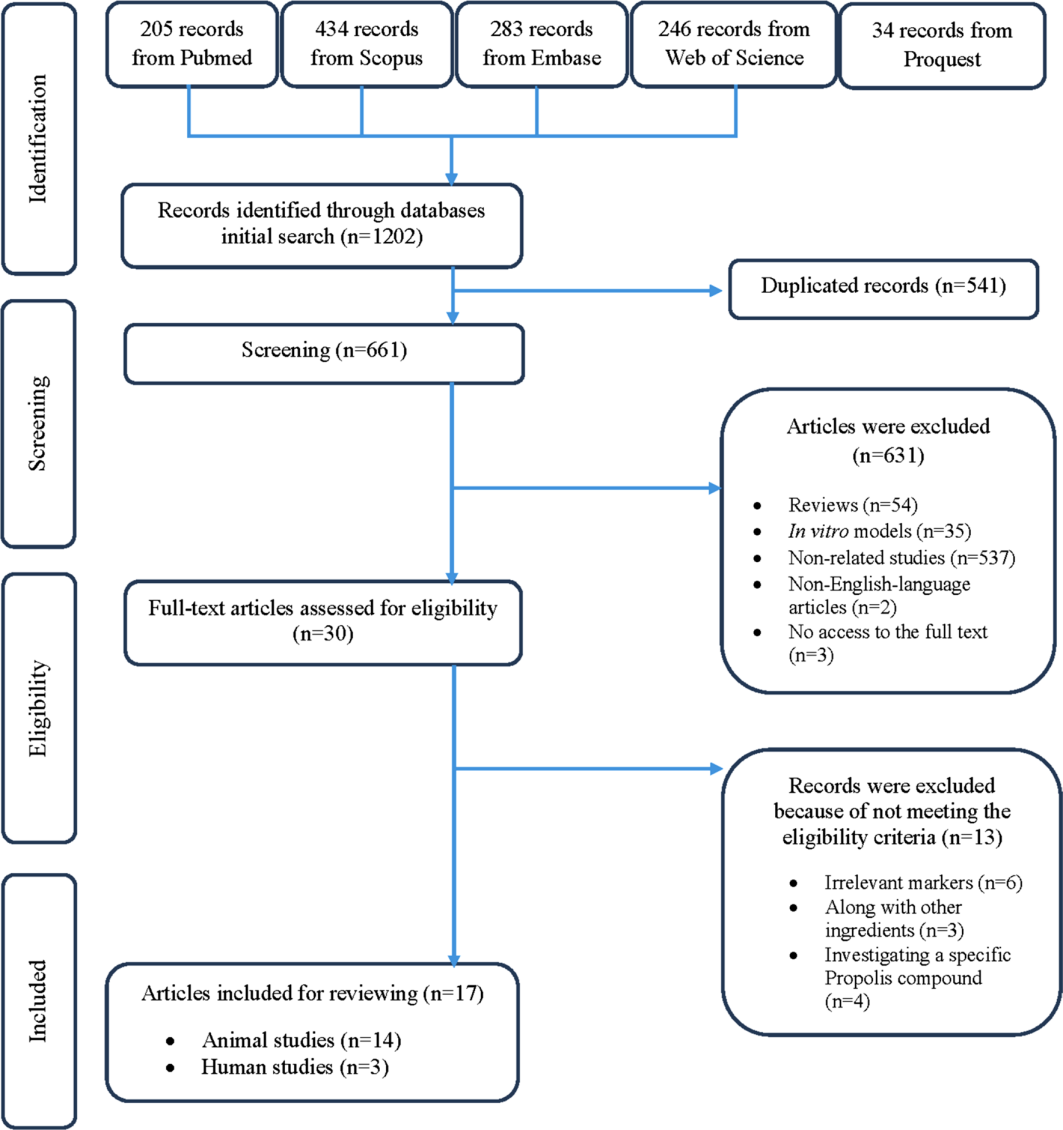
Flow diagram of the literature search and study selection process

The flowchart of the process for selecting the studies was summarized in Fig. 1. A total number of 1202 articles were retrieved after the initial search, 541 were duplicated, and therefore 661 non-duplicated publications remained. Of these, 631 articles were excluded after checking titles and abstracts. In the next step, 13 articles were excluded due to not meeting the eligibility criteria. Finally, only 17 articles met the selection criteria and were included in this systematic review. The characteristics of the selected studies are provided in Table 1.

**Table 1:** Characteristics of studies that reported the roles of Propolis in kidney disease

Articles	Cause of AKI or CKD	Samples	Study design	Main results
*A. Animal studies*				
Laaroussi et al. [19] Morocco	T2DM: Induced by 10% D-glucose in drinking water throughout the study	Sixty-six male Wistar rats (weighing 173 ± 3 g) were randomly allocated into eleven groups of six rats (*groups 1–5: non-diabetic controls; groups 6–11: diabetics*): Group 6: untreated; Group 7: Propolis extract (200 mg/Kg); Group 8: bee pollen extract (200 mg/kg); Group 9: Propolis extract (100 mg/kg); Group 10: bee pollen extract (100 mg/kg); Group11: 100 mg/kg of Propolis extract + 100 mg/kg of bee pollen extract	Administration of Moroccan Propolis: 100 or 200 mg/kg/day, by oral gavage for 16 weeks	**Comparison between groups 7 and 9 with group 6:** **Significant decrease:** FBS, insulin, HOMA-IR, TC, TG, LDL-C, VLDL-C, SCr, urea, uric acid, kidney weight **Significant increase:** HOMA-β (only at a dose of 200 mg/kg/day), QUICKI, HDL-C, total serum protein, serum albumin **Insignificant:** Na^+^, K^+^, Cl^**−**^ *Data regarding the comparison between groups 10 and 11 were not presented in the article
El Adaouia Taleb et al. [20] Algeria	DM: Induction by i.p. injection of a single dose of STZ solution (65 mg/kg)	Twenty male Wistar rats (weighing 250–300 g) were randomly assigned into four groups of five animals each: Control group, untreated diabetic group, and two diabetic groups treated with Propolis (Group DP30% and Group DP15%)	Administration of the 30% or 15% Turkish Propolis ethanolic extract: 0.5 mL/100 g BW/day, by oral gavage for 4 weeks	**Comparison between Propolis receiving groups with untreated Diabetic group:** **Significant decrease:** FBS **Significant increase:** renal tissue improvement
El Menyiy et al. [21] Morocco	DM: Induction by a single dose intravenous injection of STZ (60 mg/kg)	Forty-eight adult male Wistar rats (weighing 150–220 g) were divided into eight groups, six rats in each (*groups 1–4 non-diabetic; groups 5–8 diabetic*): Group 5: untreated; Group 6: glibenclamide; Groups 7 and 8: Propolis (50 or 100 mg/kg/day)	Administration of hydroalcoholic extract of Moroccan Propolis: 50 or 100 mg/kg/day, by oral gavage for 15 days	**A) Comparison between Diabetes given single dose of Propolis with untreated Diabetic group:** **Significant decrease:** FBS **B) Comparison between groups 7 and 8 with group 5:** **Significant decrease:** FBS, TC, TG, LDL-C, VLDL-C, urea (only at a dose of 100 mg/kg/day), SCr **Significant increase:** HDL-C, albumin **Insignificant:** serum protein
Geyikoglu et al. [22] Turkey	I/R	Thirty-five Sprague–Dawley rats (weighing 250–300 g) were randomly divided into five groups: 1. Control -Sham group, 2. I/R group, 3. The Propolis intervention group, 4. Boric acid intervention group, 5. Propolis + boric acid intervention group (n = 7 per group)	Administration of water-soluble Propolis: 200 mg/kg intra-gastric, one hour before ischemia	**Comparison between I/R given Propolis with untreated I/R group:** **Significant decrease:** renal MDA, renal 8-OHdG formation, renal TNF-α, renal congestion, renal hemorrhage, renal hydropic degeneration, necrosis of tubules **Significant increase:** renal SOD, renal GSH, improvement of kidney tissue **Insignificant:** Bax Immunoreactivity
Salmas et al. [23] Turkey	HTN: Induction of HTN by i.p. injection of non‐specific NOS inhibitor L‐NAME (40 mg/kg) for 28 days	Thirty-five male Sprague–Dawley rats (weighing 250–300 g) were separated into five groups of 7 rats each: Group I (control), Group II (L‐NAME = HT), Group III (L-NAME + Propolis), Group IV (L‐NAME + CAPE), and Group V (L‐NAME + pollen)	Administration of Propolis: 200 mg/kg/day, by gavage for 2 weeks	**Comparison between L-NAME given Propolis with untreated L-NAME group:** **Significant decrease:** renal TOS, renal OSI, renal NF-κB, renal ADMA **Significant increase:** renal TAS, renal PON1
El Rabey et al. [24] Saudi Arabia	DM: Induction of DM by intravenous injection of STZ 65 mg/kg	Forty male Albino rats (weighing 180–200 g) were separated into four groups (10 rats in each group): negative control (G1), positive diabetic control (G2), the Nigella sativa group (G3), and the Propolis group (G4)	Administration of Saudi Arabian Propolis methanol extract: 20% w/w (200 g of Propolis in one liter of methanol; 20 g of it in 100 ml distilled water with 2 mL of tween 80 (suspending agent) to prepare a 20% solution), orally, for 4 weeks	**Comparison between Diabetes given Propolis with untreated Diabetic group:** **Significant decrease:** FBS, percentage of CML, serum and renal MDA, serum and renal IL-6, serum IgG, serum IgA, serum IgM, UAE, urea, SCr, uric acid **Significant increase:** Serum CAT, serum SOD, serum GST, improvement of the kidney tissue, urine Cr, serum electrolytes levels (Na^+^ and K^+^)
Sameni et al. [25] Iran	T1DM: Induction of T1DM by a single dose of STZ 60 mg/kg by i.p. injection	Forty male Wistar rats (weighing 200–220 g) were divided into five groups (8 rats per group): control, untreated DM, DM with vehicle treatment (10% ethanol), DP100, and DP200	DP100: DM with administration of 100 mg/kg/day ethanol extract of Iranian Propolis DP200: DM with administration of 200 mg/kg/day ethanol extract of Iranian Propolis All by oral gavage, for 6 weeks	**Comparison between Diabetes given Propolis with untreated or vehicle-treated Diabetes:** **Significant decrease:** In both groups: FBS, GBM thickness, In the DP200 group: renal MDA, kidney weight, GA **Significant increase:** In both groups: renal SOD, renal GPx In the DP200 group: renal FRAP **Insignificant:** In the DP100 group: renal MDA, renal FRAP, kidney weight *With adjusting blood glucose level, the significance for GA, GBM, MDA, FRAP and GPx (except SOD) were impaired
Jabir et al. [26] IRAQ	DM: Stimulating diabetes in fasting rats by injection of a single dose of STZ (i.p.) concentration of 60 mg/kg for more than sixteen hours	Seventy-five Sprague–Dawley rats (weighing 150 ± 10 g) were divided randomly into five groups: (1) control (intact rats drenched orally with drinking water), (2) diabetic rats (rats were drenched drinking water), (3) EEP treated intact rats, (4) diabetic with pre-treated of EEP, 5. Post-treated of EEP in diabetic rats (n = 15 per group)	Administration of ethanol extract of Iraqi Propolis: 200 mg/kg/day by drenching for three or 6 weeks	**Comparison between EEP pre-treated and Post-treated diabetic rats with untreated Diabetic group:** **Significant decrease:** FBS, serum MDA, serum NO, uric acid **Significant increase:** serum SOD, serum CAT, serum GST, improvement of the renal tissue, concentration of total serum protein
Teles et al. [10] Brazil	5/6 renal ablation (Nx)	Thirty-two adult male Wistar rats (aged 2 months, weighing 220–250 g) were divided into five groups: Sham (n = 8) and Nx (n = 11) untreated rats; Sham + RP (n = 8) and Nx + RP (n = 8) treated rats after 30 days of surgery	Administration of alcoholic extract of Brazilian RP: 150 mg/kg/day orally, for 2 months	**Comparison between Nx given RP with untreated Nx group:** **Significant decrease:** Tbars, renal ED-1^+^ cells, GS, IG, INT, proteinuria, SCr, HTN **Significant increase:** improvement of renal damage **Insignificant:** renal AII^+^ cells
da Costa et al. [27] Brazil	Unilateral nephrectomy and contralateral renal I/R	Thirty-two male Wistar Rats (weighing 250–300 g) were divided into four groups: 1. Sham + tap water, 2. Sham + RP, 3. I/R + tap water, 4. I/R + RP (n = 8 in each group)	Administration of RP: 150 mg/kg/day by gastric gavage, 3 days before the procedure, and one hour prior to surgical procedure or ischemia	**Comparison between I/R given RP with untreated I/R group:** **Significant decrease:** urine MDA, renal MDA, tubular necrosis score, urea, SCr, FENa^+^, FEK^+^, **Significant increase:** renal GSH, renal eNOS score, renal HO-1 score, improvement of kidney damage, ClCr
Oršolić et al. [28] Croatia	DM: Induction of DM by a single intravenous injection of alloxan 75 mg/kg	Seventy male and female Swiss Albino mice (2–3 months old, weighing 20–25 g) were divided into four groups: control, alloxan control, WSDP-treated diabetic group, EEP –treated diabetic	Administration of Croatian WSDP: 50 mg/kg/day Administration of Croatian EEP: 50 mg/kg/day All by i.p. injection for 7 days	**Comparison between Diabetes given Propolis with untreated Diabetic group:** **Significant decrease:** In both groups: liver MDA By WSDP: renal MDA **Insignificant:** In both groups: FBS, TG, TC, improvement of renal histopathology By WSDP: urea (decreased) By EEP: urea (increased), renal MDA
Zhu et al. [29, 30] China	T1DM: Induction of T1DM by intravenously injection of a single dose of STZ 50 mg/kg	Forty male Sprague–Dawley rats (weighing 270 ± 40 g) were divided into the following groups: 8 healthy rats as normal group and 32 diabetic rats in four groups of model (untreated diabetes), Chinese Propolis, Brazilian Propolis, and positive (10 mg/kg glucobay)	Chinese group: Administration of 100 mg/kg/day ethanol extracted Chinese Propolis Brazilian group: Administration of 100 mg/kg/day ethanol extracted Brazilian Propolis All by oral intubation, twice daily, for 8 weeks	**Comparison between Diabetes given Propolis with untreated Diabetic group:** **Significant decrease:** In both groups: FBS, renal MDA, UAER Chinese group: HbA1C, TC, serum MDA Brazilian group: serum NOS, liver MDA, BUN **Significant increase:** In both groups: renal CAT In Brazilian group: serum and liver SOD, liver GPx **Insignificant:** In both groups: TG, LDL-C, HDL-C, serum NO, serum and renal GPx, serum and liver CAT, renal SOD, CCR, SCr, kidney weight In Chinese group: liver MDA, liver GPx, serum and liver SOD, serum NOS, BUN In Brazilian group: HbA1C, TC, serum MDA
Zhu et al. [29, 30] China	DM (STZ-induced hepatorenal injury): Induction of diabetes by intravenously injection of STZ 50 mg/kg	Forty male Sprague–Dawley rats (5 weeks old, weighing 230–280 g) were divided into the following groups: 8 healthy rats as normal group and 32 diabetic rats in four groups of model (untreated diabetes), A, B, and positive (10 mg/kg glucobay)	Group A: Administration of 100 mg/kg/day ethanol extracted Chinese Propolis Group B: Administration of 100 mg/kg/day ethanol extracted Brazilian Propolis All by oral intubation, twice daily, continuously for 8 weeks	**Comparison between Diabetes given Propolis with untreated Diabetic group:** **Significant decrease:** In both groups: renal GPx, renal MDA, UAER, microalbuminuria, In group A: HbAlC In group B: serum and hepatic MDA, serum NOS **Significant increase:** In both groups: serum SOD, hepatic GPx, kidney tissue health (a better in kidney lesions in group A than group B) In group B: renal CAT improvement **Insignificant:** In both groups: serum NO, serum CAT, serum GPx, liver and renal SOD, liver CAT, CCR, BUN, SCr In group A: serum NOS, serum and hepatic MDA, renal CAT In group B: HbA1C
Abo-Salem et al. [31] Egypt	T1DM: Induction of T1DM by i.p. injection of STZ 60 mg/kg for three successive days	Fifty adult male Albino Wistar rats (weighing 190–200 g) were divided into five groups (10 animals/ group): control (G1), diabetic control (G2), three groups of EEP (G3, G4, G5)	Administration of ethanol extract of Brazilian green Propolis: 100, 200, 300 mg/kg/day, via oral gavage, for 40 days	**Comparison between Diabetes given Propolis with untreated Diabetic group:** **Significant decrease:** FBS, TC, LDL-C, TG, serum and renal MDA, kidney weight, UAE, BUN, SCr (at doses of 200 and 300 mg/kg) **Significant increase:** HDL-C (at doses of 200 and 300 mg/kg), renal GSH, renal SOD, renal CAT **Insignificant:** HDL-C (at dose of 100 mg/kg), SCr (at dose of 100 mg/kg),
B. Human studies (RCTs)*				
Silveira et al. [13] Brazil	CKD caused by diabetes or another etiology	Thirty-two CKD patients were randomized to receive Brazilian green Propolis extract (n = 18) or a placebo (n = 14)	Supplementation with Propolis 500 mg/day (4 tablets of 125 mg each) or placebo 500 mg/day (4 tablets of 125 mg each), for 12 months	**Comparison between patients given Propolis with the placebo group:** **Significant decrease:** urinary MCP-1, proteinuria **Insignificant:** HbA1C, eGFR, UACR, BP
Zakerkish et al. [32] Iran	T2DM	Ninety-four Patients with T2DM (35–85 years old, receiving treatment with oral hypoglycemic agents) were randomized to receive: Iranian Propolis (n = 50) or placebo (n = 44)	Supplementation with ethanol extract of Iranian Propolis 1000 mg/day (2 capsules of 500 mg each) or placebo 1000 mg/day, for 90 days	**Comparison between patients given Propolis with the placebo group:** **Significant decrease:** HbA1C, 2hpp Glc, insulin, HOMA-IR, HOMA-β, serum hs-CRP, serum TNF-α **Significant increase:** HDL-C **Insignificant:** FBS, TG, TC, LDL-C, VLDL-C, serum IL-6, serum IL-1β, eGFR, BUN, SCr, uric acid
Fukuda et al. [33] Japan	T2DM	Eighty patients with T2DM were randomly assigned to receive Brazilian green Propolis (n = 41) or the placebo (n = 39)	Supplementation with Brazilian green Propolis or placebo 226.8 mg/day, once a day for 8 weeks	**Comparison between patients given Propolis with placebo group:** **Insignificant:** FBS, HbA1C, insulin, HOMA-IR, TC, TG, HDL-C, LDL-C, RLP-C, serum TNF‑α, serum IL-6, serum hs-CRP, eGFR, UACR, uric acid, urine pH *Values of blood uric acid and eGFR in patients taking the placebo became worse at 8 weeks compared to the baseline, whereas this did not occur in patients consuming Brazilian green Propolis

Bold used to make it easy distinguishing the results of different groups

AKI, acute kidney injury; CKD, chronic kidney disease; T2DM, type 2 diabetes mellitus; g. gram; mg, milligram; kg, kilogram; FBS, fasting blood sugar; HOMA‑IR, homeostasis model assessment of insulin resistance; TC, total cholesterol; TG, triglyceride; LDL-C, low-density lipoprotein cholesterol; VLDL-C, very low-density lipoprotein cholesterol; SCr, serum creatinine; HOMA-β, homeostasis model assessment of β-cell function; QUICKI, quantitative insulin sensitivity check index; HDL-C, high-density lipoprotein cholesterol; Na^+^, Sodium; K^+^, Potassium; Cl^−^, Chloride; DM, diabetes mellitus; i.p., intraperitoneally; STZ, streptozotocin; mL, milliliter; BW, body weight; I/R, ischemic-reperfusion; n, number; MDA, malondialdehyde; 8-OHdG, 8-hydroxy-2′-deoxyguanosine; TNF-α, tumor necrosis factor α; SOD, superoxide dismutase; GSH, glutathione; HTN, hypertension; NOS, nitric oxide synthetase; L‐NAME, Nω‐nitro‐L‐arginine methyl ester; HT, hypertensive; CAPE, caffeic acid phenethyl ester; TOS, total oxidant status; OSI, oxidative stress index; NF‐κB, nuclear factor kappa B; ADMA, asymmetric dimethylarginine; TAS, total antioxidant status; PON1, paraoxonase; CML, Carboxymethyl Lysine; IL-6, interleukin-6; Ig, Immunoglobulin; UAE, urinary albumin excretion; CAT, catalase; GST, glutathione-S-transferase; Cr, creatinine; T1DM, type 1 diabetes mellitus; GBM, glomerular basement membrane; GA, glomerular area; GPx, glutathione peroxidase; FRAP, ferric-reducing ability of plasma; EEP, ethanolic extract of Propolis; NO, nitric oxide; Nx, 5/6 renal ablation; RP, red Propolis; Tbars, urinary levels of reactive oxygen metabolites; ED-1, interstitial and glomerular macrophage infiltration; GS, percentage of Sclerotic glomeruli; IG, glomerulosclerosis Index; INT, masson positive cortical interstitial area; AII, angiotensin II; FENa^+^, absolute excretion of sodium; FEK^+^, absolute excretion of potassium; eNOS, endothelial nitric oxide synthetase; HO-1, heme-oxygenase-1; ClCr, creatinine clearance; WSDP, water-soluble derivative of Propolis; UAER, urinary albumin excretion rate; HbA1C, hemoglobin A1C; BUN, blood urea nitrogen; CCR, creatinine clearance rate; MCP-1, monocyte chemoattractant protein-1; eGFR, estimated glomerular filtration rate; UACR, urinary albumin-to-creatinine ratio; BP, blood pressure; 2hpp Glc, 2-h postprandial glucose; hs-CRP, high sensitivity C-reactive protein; IL-1 β, interleukin-1β; RLP-C, remnant-like particle cholesterol

*RCT: Randomized clinical trials

### Overview of Propolis

#### Composition and characterization of Propolis

Propolis, commonly known as the “bee glue,” is the third most important part of bee products [15, 17]. Propolis is a plant derived substance that honeybees make it by mixing their saliva containing specific enzymes and beeswax with exudate collected mostly from leaf and flower stems, buds, and bark cracks of various species of trees, and it contains multiple polyphenolic compounds, mostly flavonoids and phenolic acids [14, 15]. The word Propolis is made up of two Greek words, pro and polis, which mean “defense” and “city” or “community,” respectively [15]. Propolis is used in the structure and maintenance of beehives as the defense mechanism [14]. Bees use it to smoother the inner surface, seal cracks and holes, maintain the internal temperature of the beehive, and prevent weathering and predator's attack. Moreover, due to its antimicrobial property, the internal environment remains aseptic [15].

Due to its main color, Propolis is divided into three classes, including green, red, and brown, with a melting point of around 65 °C, but in some samples, its melting point goes higher, up to 100 °C [14, 18]. It is a resinous product with more than 300 compounds that vary based on types of hives, geographical origins, and seasons [14, 15], with some changes in its compounds profile due to extracting with water or ethanol [14]. Propolis compounds include phenolic acids, flavonoids, esters, diterpenes, sesquiterpenes, aromatic aldehydes, lignans, alcohols, amino acids, fatty acids, vitamins (thiamin, riboflavin, pyridoxine, C, and E), and minerals [14, 15]. The pharmacological properties of flavonoids are mostly due to their structural features as tricyclic compounds, resulting in attaching free radicals to their rings. The polyphenolic amount of different Propolis samples differs significantly, ranging from 143 to 324 mg gallic acid equivalents/g and from 206 to 705 mg quercetin equivalents/g of ethanolic extracts of Propolis (EEP), respectively. The phenolic content of Propolis, based on high-performance liquid chromatography (HPLC) analysis, commonly consists of chrysin, galangin, pinobanksin, pinostrobin, and pinocembrin, the last being the most abundant flavonoid in Propolis [14].

#### Bioavailability of Propolis

Propolis absorption and bioavailability is low due to its structure containing lipids, waxes, and resins in a complex substrate with a high molecular weight. The presence of various polyphenols with synergistic effects and forms used (natural fruit, juice, or extract) are essential contributors of bioavailability [34]. Poor bioavailability of polyphenols could be because of digestive instability, low transcellular efflux in intestinal cells, and rapid metabolism and excretion [35]. The conversion of the polyphenolic compounds, which are poorly bioavailable, to the smaller phenolic acids with increased bioavailability by the colonic microbiota and intestinal enzymes is an essential contributor in the beneficial effects of these compounds, and as gut microbiota varies between people, the absorption and metabolism differ individually [36, 37]. Due to the high initial contents of phenolic compounds in Propolis compared to fruits and vegetables, its detected plasma levels were still high despite the low absorption rate [37]. The therapeutic effects of Propolis phenols in the bloodstream are restricted by their selective permeability across the blood–brain barrier and systemic elimination [34]. However, a recent study pointed that caffeic acid phenethyl ester (CAPE), a component found in Propolis, can cross the blood–brain barrier in rats [38]. It was reported that CAPE undergoes hydrolysis to caffeic acid within six hours of reaching the rat plasma [39]. Because of the lack of carboxylesterase enzyme in human plasma, which may be responsible for CAPE's hydrolysis, this type of conversion does not occur in humans [40]. Lipophilicity of Propolis polyphenols and metabolized derivatives is an important indicator of their permeability across the blood–brain barrier, in a way that less polar polyphenols and/or metabolites (i.e., O-methylated derivatives) have greater brain uptake compare to more polar ones (i.e., sulfated and glucuronidated derivatives) [41]. Polyphenols excretion in the urine differs individually, which may be related to aging, kidney function, or Propolis properties [14]. Continuing studies on the general health of Propolis consumers and the effects of this compound on renal function are necessary.

#### Biological activities of Propolis

Propolis has attracted attention in recent years because of its potential reported benefits in preventing and treating diseases, and a number of scientific articles have been widely investigated the bioactivity and health benefits of Propolis [14]. Anti-inflammatory, antioxidant, antiseptic, and immunomodulatory activities of Propolis, as well as its role in prevention and control of neoplastic disorders, and some chronic diseases like diabetes, atherosclerosis, HTN, neurodegenerative disorders, dental caries, and liver and kidney diseases, has probably come from the existing bioactive phytochemicals constituents and made it a valuable point for research [14–18]. The attenuation of adverse effects of the chemotherapeutic agents has been mentioned as another property for Propolis [15, 16]. Notably, the Propolis type and the solvents used for its extraction determine the biological activity of this material [16]. Also, Propolis polyphenols have recently been defined as probiotics by an international consortium due to their selective metabolism by gut microbiota. Propolis polyphenols inhibit the growth of pathogenic bacteria and suppress gut pathogens' adherence to human gut cells, so it may improve gut health [14]. Additionally, due to antimicrobial (the most extensively reported property of Propolis), antiseptic, anti-inflammatory, antioxidant, and immunomodulatory effects of Propolis, it has been widely used as an external treatment for wounds and burns. These properties perhaps increase cell proliferation in the skin and activate the remodeling of the skin tissue [16]. Propolis was also researched in cosmetic industries. The studies reported that it could act as a sunscreen agent; therefore, it can be used as an ingredient of sunscreen cosmetics [15]. The Propolis trade is growing, and it is found commercially in the forms of lozenges, mouthwash, toothpaste, creams, dentifrices, cough syrups, gels, wine, powder, cake, soap, chewing gums, and tablets [14, 42]. Sales on Propolis containing products for oral health and wound care appear at the top of the category list [14].

#### Side effects and toxic properties of Propolis

Clinical studies in mice and humans show that Propolis and its compounds are usually well-tolerated and non-toxic when used in moderation [43–45]. However, it must be reminded that few human trials include side effects and toxicity of Propolis as an outcome measure [14].

In a reported case, a 59-year-old man with cholangiocarcinoma who was supplementing with Propolis developed AKI, and his kidney function improved after hemodialysis and withdrawal of Propolis. However, he continued the consumption of Propolis for his cancer because he was uncertain about the role of the Propolis in the development of AKI. Renal function worsened again and improved with discontinuation. Renal biopsy was not taken from the patient in this study; however, the likely side effects of the Propolis became a topic of interest. The probable mechanism proposed in this study was the CAPE mediated inhibition of cyclooxygenase and inducible nitric oxide synthase (iNOS) pathways as it happens in nonsteroidal anti-inflammatory drug-related AKI [46].

Based on previous animal studies, the safe concentration of Propolis for generally healthy humans is approximately 1.4 mg/kg/day or 70 mg/day [47]. Interestingly, studies have concluded that even using 150 mg of pinocembrin, a component of Propolis as a single dose, is safe [45]. The median lethal dose (LD50) of Propolis extract while given to mice is higher than 7.34 g/kg, assures human therapeutic dosage safety [48, 49]. However, determining the proper dose of Propolis because of the different studied populations, dosing regimens, patient's compliance, and purity of the product is difficult. Also, differences in Propolis's phenolic compounds and their bioactivity due to different geographical areas make it difficult to determine the exact appropriate dosage [14]. Hypersensitivity is the more common adverse effect of Propolis, especially in topical use, which causes allergic reactions, swelling, dermatitis, and urticaria [50]. Dermatitis induced by Propolis was first reported by beekeepers; over time, as the usage of Propolis developed, non-occupational cases were also reported [51]. 1.2–6.6% of individuals with dermatitis were sensitive to Propolis [52]. Allergy to Propolis is more common in children; therefore, patch warnings were advised for dermatological use in young children [53–55].

### Propolis and metabolic variables (glycemic and lipid profile) in kidney disease

#### Glycemic profile

##### Animal studies

The effects of Propolis supplementation on glycemic parameters have been investigated in ten among the fourteen animal studies. In a study conducted by Laaroussi et al., it was revealed that Moroccan Propolis administration (100 or 200 mg/kg/day) to diabetic rats for 16 weeks resulted in significantly decreased fasting blood sugar (FBS), serum insulin, and homeostasis model assessment of insulin resistance (HOMA-IR), and increased homeostasis model assessment of β-cell function (HOMA-β) (only at a dose of 200 mg/kg/day) and quantitative insulin sensitivity check index (QUICKI) [19]. In another study by El Adaouia Taleb et al., it was reported that administration of 30% or 15% Turkish Propolis ethanolic extract at the dosage of 0.5 ml/100 g BW/day in diabetic rats for 4 weeks significantly lowered FBS levels, while the rate in 30% propolis treated group had normalized [20]. Besides, in El Menyiy et al. experimental study, 50 or 100 mg/kg/day hydroalcoholic extract of Moroccan Propolis was administered to diabetic rats and the levels of FBS one, two, and three hours, as well as 15 days after first administration, were measured. It was shown that Propolis at both doses could significantly decrease the FBS levels, and it was more potent than glibenclamide at an amount of 100 mg/kg/day [21]. In a study by Rabey et al., administration of Propolis methanol extract (20% w/w) to diabetic rats for 4 weeks resulted in a significant reduction of FBS and percentage of carboxymethyl lysine (CML), as a marker of advanced glycation end products (AGEs) [24]. Moreover, Sameni et al. reported a significantly reduced FBS after 6 weeks of 100 and 200 mg/kg/day EEP administration in diabetic rats [25]. In another study, receiving 200 mg/kg/day EEP for 3 weeks in two groups of rats, one group before induction of diabetes and another group after induction, showed a significant reduction in FBS, and it was more pronounced in the treatment group than the pre-DM-induction group [26]. Zhu et al. administered 100 mg/kg/day ethanol extracted Chinese or Brazilian Propolis for 8 weeks to diabetic rats and reported that the intervention could reduce FBS in both groups except the hemoglobin A1C (HbA1C) that was only decreased in the Chinese Propolis recipient group [29]. Another study by Zhu et al., with the same dosage and duration of supplementation in diabetic rats, showed significant reductions in HbA1C only in the Chinese Propolis group [30]. In another study by Abo-Salem et al., 100, 200, 300 mg/kg/day ethanol extract of green Propolis administration for 40 days significantly decreased FBS in all supplementation dosages in diabetic rats [31]. However, Orsolic et al. showed that in diabetic mice fed 50 mg/kg/day water or ethanol extract of Propolis for 1 week, FBS alterations were not considerable in both groups [28].

##### Human studies

All three human studies included in this article have reported the effects of Propolis supplementation on glycemic parameters. In the study by Fukuda et al., green Propolis supplementation with a dose of 226.8 mg/day for 8 weeks in patients with type 2 diabetes did not make any significant changes in HOMA-IR, FBS, HbA1c, and insulin levels [33]. In another study, Silveira et al. conducted a double-blind, placebo-controlled clinical trial on CKD patients and reported that Propolis did not result in any significant changes in HbA1c following green Propolis supplementation with a 500 mg/day dose for 1 year [13]. However, Zakerkish et al. showed that Propolis supplementation of 1000 mg/day for 90 days in T2DM patients could significantly decrease HbA1C, 2-h postprandial glucose (2hpp Glc), insulin, HOMA-IR, and HOMA-β levels but has no significant effect on FBS concentrations [32].

#### Lipid profile

##### Animal studies

The effects of Propolis on lipid profile have been reported in five out of the fourteen animal models of kidney disease studies. In a study by Laaroussi et al. on diabetic rats, 100 or 200 mg/kg/day Moroccan Propolis administration for 16 weeks resulted in a significant decrease in total cholesterol (TC), triglyceride (TG), low-density lipoprotein cholesterol (LDL-C), and very low-density lipoprotein cholesterol (VLDL-C), and increase in high-density lipoprotein cholesterol (HDL-C) levels [19]. Similarly, in another study by El Menyiy et al., the significant decrease in terms of TC, TG, LDL-C, and VLDL-C and increase in HDL-C levels were reported in diabetic rats receiving 50 or 100 mg/kg/day hydroalcoholic extract of Moroccan Propolis for 15 days [21]. Zhu et al. administered 100 mg/kg/day ethanol extracted Chinese or Brazilian Propolis to diabetic rats for 8 weeks and reported that the intervention could reduce serum TC levels in only the Chinese Propolis group but had not any significant effects on serum concentrations of TG, LDL-C, and HDL-C in both groups [29]. Also, Abo-Salem et al. reported that the administration of EEP (100, 200, and 300 mg/kg/day) to diabetic rats for 40 days significantly reduced serum TC, LDL-C, and TG levels in all doses, and significantly increased HDL-C level at the doses of 200 and 300 mg/kg/day [31]. In contrast, Orsolic et al. reported that water and ethanol extracted Propolis given at the dose of 50 mg/kg/day to diabetic rats for seven days did not change serum TG and TC levels, the result was independent of the Propolis preparation methods [28].

##### Human studies

Among the three selected human studies, two of them assessed the effectiveness of Propolis on dyslipidemia. In a trial by Zakerkish et al., the levels of HDL-C significantly increased in diabetic patients following Propolis intake of 1000 mg/day for 90 days; however, the serum levels of TG, TC, LDL-C, and VLDL-C did not show any significant changes in their reports [32]. In another study involving diabetic patients, Fukuda et al. reported that 226.8 mg/day Brazilian green Propolis for 8 weeks did not improve lipid profile (serum levels of TG, TC, LDL-C, HDL-C, and remnant-like particle cholesterol (RLP-C)) significantly [33].

### Propolis and oxidative stress indices in kidney disease

#### Animal studies

Eleven out of fourteen animal studies included in this article have evaluated the possible effects of Propolis on oxidative parameters. In a study by Rabey et al., the administration of Propolis methanol extract (20% w/w) to diabetic rats for 4 weeks resulted in a significant reduction of serum and renal tissue malondialdehyde (MDA) and a significant increase of serum catalase (CAT), superoxide dismutase (SOD), and glutathione-S- transferase (GST) [24]. Orsolic et al. showed that in diabetic mice fed by 50 mg/kg/day water or ethanol extract of Propolis for 1 week, liver MDA levels in both groups and renal MDA only in the water extract group significantly decreased [28]. Zhu et al. administered 100 mg/kg/day ethanol extracted Chinese or Brazilian Propolis for 8 weeks to diabetic rats, and reported that the intervention could reduce renal MDA in both groups, serum MDA in the Chinese Propolis group, and serum nitric oxide synthetase (NOS) and liver MDA in the Brazilian Propolis group, and also could increase renal CAT in both groups and serum and liver SOD and liver glutathione peroxidase (GPx) in Brazilian Propolis group. Despite these, they did not see any significant effects on serum nitric oxide (NO), serum and renal GPx, serum and liver CAT, and renal SOD alterations in this study [29]. In another study done by Zhu et al., with the same dosage and duration of supplementation in diabetic rats, there were significant reductions in renal GPx and MDA in both groups and serum and hepatic MDA and serum NOS only in the Brazilian Propolis group, a significant increase in serum SOD and hepatic GPx in both groups, and renal CAT only in Brazilian Propolis group, while alterations of serum NO, serum CAT, serum GPx, liver and renal SOD, and liver CAT were insignificant [30]. In another study by da Costa et al., involving rats exposed to unilateral nephrectomy and contralateral renal ischemic-reperfusion (I/R), administration of 150 mg/kg/day of red Propolis (RP) 3 days before the procedure and one hour prior to surgical procedure or ischemia caused a significant decrease in urine and renal tissue MDA and a significant increase in renal tissue glutathione (GSH), renal endothelial NOS (eNOS) score, and renal heme-oxygenase-1 (HO-1) score [27]. Also, Teles et al. reported that in 5/6 renal removed rats, administration of 150 mg/kg/day alcoholic extract of RP for 2 months led to a significant decrease in urinary levels of reactive oxygen metabolites (T-bars) [10]. Salmas et al. found out that in hypertensive rats, administration of 200 mg/kg/day Propolis for 2 weeks led to a significant decrease in renal tissue total oxidant status (TOS) and oxidative stress index (OSI), as well as a significant increase of total antioxidant status (TAS) and paraoxonase (PON1) [23]. Moreover, Geyikoglu et al. discovered significantly decreased renal tissue MDA and 8-hydroxy-2′-deoxyguanosine (8-OHdG) formation in rat kidney cells and a considerably increased renal tissue SOD and GSH after 200 mg/kg administration of water-soluble Propolis one hour before ischemia in rats exposed to I/R [22]. Sameni et al. administered 100 and 200 mg/kg/day EEP for 6 weeks to diabetic rats and reported that the intervention could significantly increase renal tissue SOD and GPx in both groups, and ferric-reducing ability of plasma (FRAP) only by 200 mg/kg dosage, and reduce renal tissue MDA again in only 200 mg/kg dosage [25]. Also, in a study by Abo-Salem et al., 100, 200, and 300 mg/kg/day ethanol extract of green Propolis administration for 40 days significantly decreased serum and renal tissue MDA, and increased renal tissue GSH, SOD, and CAT in all dosages of supplementation in diabetic rats [31]. In another study, receiving 200 mg/kg/day EEP for 3 weeks in two groups of rats, one group before induction of diabetes and another group after induction of it, showed significant reductions in serum MDA and NO, and significant elevation in serum SOD, CAT, and GST concentrations [26].

### Propolis and inflammation biomarkers in kidney disease

#### Animal studies

Four of the fourteen animal studies assessed the effects of Propolis on inflammation status. Salmas et al. reported that administering 200 mg/kg/day Propolis for 2 weeks in hypertensive rats led to a significant decrease in renal levels of nuclear factor kappa B (NF‐κB) [23]. Moreover, Geyikoglu et al. observed significantly decreased renal levels of tumor necrosis factor α (TNF-α) after administration of 200 mg/kg water-soluble Propolis one hour before ischemia in rats exposed to I/R [22]. Also, in Rabey et al. study, supplementing diabetic rats with Propolis methanol extract (20% w/w) for 4 weeks resulted in a significant reduction in the levels of serum and renal interleukin-6 (IL-6) and serum immunoglobulins (IgG, IgA, and IgM) [24]. Similarly, in a study by Teles et al. on rats with 5/6 renal ablation, alcoholic extracted RP intake of 150 mg/kg/day for 2 months significantly decreased renal tissue inflammation (interstitial and glomerular macrophage infiltration; as ED-1^**+**^ cells) [10].

#### Human studies

The effects of Propolis on inflammation biomarkers in kidney disease were investigated in all three human studies. In a randomized controlled trial by Silveira et al., Brazilian green Propolis supplementation at the dosage of 500 mg/day in CKD patients for 12 months significantly decreased urinary monocyte chemoattractant protein-1 (MCP-1) levels [13]. Similarly, in Zakerkish et al. study, Propolis intake at the dosage of 1000 mg/day in patients with T2DM for 90 days significantly caused serum reduction of high sensitivity C-reactive protein (hs-CRP) and TNF-α levels but did not significantly change serum levels of interleukin-1β (IL-1β) and IL-6 [32]. Conversely, Fukuda et al. reported that administration of Brazilian green Propolis at the dosage of 226.8 mg/day in diabetic patients for 8 weeks did not change the serum levels of TNF-α, IL-6, and hs-CRP remarkably [33].

### Propolis and renal damage morphology and structure in kidney disease

#### Animal studies

Twelve out of the fourteen animal studies evaluated the potential effects of Propolis on the morphology and structure of kidney. In a study conducted by El Adaouia Taleb et al., the histopathological assessment indicated the DN manifestations in untreated diabetic rats, including mesangial expansion, glomerulosclerosis (GS), and tubular atrophy. By administering 0.5 ml/100 g BW/day of 30% or 15% Turkish Propolis ethanolic extract for 4 weeks, totally healthy tubules, as well as fewer glomeruli at the mesangial expansion and GS stages, were observed. The 30% Propolis was more effective than the 15% in preserving renal glomeruli [20]. In Geyikoglu et al. study, pretreatment with 200 mg/kg water-soluble extract of Propolis in rats with renal I/R injury one hour before ischemia significantly decreased the renal congestion, renal hemorrhage, renal hydropic degeneration, and tubular necrosis; however, the number of Bax-positive (a pro-apoptotic protein) cells did not change significantly [22]. Also, Jabir et al. assessed the effects of 200 mg/kg/day EEP in diabetic rats for 3 weeks. In the untreated diabetic rats, renal histopathological changes were reported as follows: severe vascular congestion, atrophy, and destruction, infiltration of red blood cells into the interstitium and tubules, presence of edematous and inflamed cells, perivascular tissue necrosis, and mild to moderate hyaline degeneration. In the diabetic rats pretreated with EEP (before streptozotocin (STZ) injection), Propolis improved the histopathological changes, as kidneys had mild to moderate vascular congestion and low infiltration of red blood cells into the interstitium, and Bowman's capsules were preserved. In the diabetic rats post-treated with EEP (after STZ injection), the assessment showed mild vascular congestion and tubular dilation, preserved Bowman's capsule, and restored renal tissue near-normal conditions; so in conclusion, Propolis improved the health and integrity of the kidney tissue [26]. In another experimental study, Rabey et al. examined the effects of methanolic extracted Propolis (20% w/w) on renal tissue for 4 weeks. In the control diabetic group, the observed pathological change in kidney structure was collapsed glomerular tuft with marked tubular atrophy, associated with interstitial inflammation and hemorrhage. Treating these diabetic rats with Propolis caused the restoration of most of the histopathologic changes in the kidney tissue nearly normal [24]. In another study by Zhu et al., there were increased volume and proliferation of mesangial cells in the glomeruli and vacuolization of renal tubular epithelial cells and casts in control diabetic rats. There was only the proliferation of mesangial cells in the glomeruli in the Chinese Propolis receiving group; in addition to this change, vacuolization of renal tubular epithelial cells was also observed in the kidneys of the Brazilian Propolis group. Overall, kidney health was significantly increased with the administration of 100 mg/kg/day of ethanol extracted Chinese or Brazilian Propolis for 8 weeks in this study; however, Chinese Propolis could improve kidney injuries better than the Brazilian one [30]. In Sameni et al. study, an intake of 100 or 200 mg/kg/day EEP in diabetic rats for 6 weeks significantly decreased glomerular basement membrane (GBM) thickness. Besides, Propolis at the dose of 200 mg/kg/day caused a significant reduction in kidney weight and glomerular area (GA) [25]. The kidney weight changes in diabetic rats were also investigated in three other studies. Similarly, in the Abo-Salem et al. study, administration of EEP (100, 200, and 300 mg/kg/day) for 40 days significantly inhibited kidney enlargement [31]. Furthermore, in a study by Laaroussi et al., with administering 100 or 200 mg/kg/day Moroccan Propolis to diabetic rats for 16 weeks, the kidney weight decreased [19]. Moreover, in Zhu et al. study, Chinese or Brazilian EEP at the dose of 100 mg/kg/day for 8 weeks did not affect kidney weight [29]. Effects of RP at a dose of 150 mg/kg/day in experimental models of CKD were investigated in two studies. Teles et al. reported that alcoholic extract of RP treatment for 2 months significantly decreased the percentage of GS (%GS), GS Index (IG), and Masson positive cortical interstitial area (as a marker of renal fibrosis) in rats with 5/6 renal ablation. As a result, RP treatment recovered the renal structural deterioration in experimental models with nephropathy [10]. Similarly, in da Costa et al. experimental study, assessment of the renal tissue showed considerable structural damages in rats exposed to unilateral nephrectomy and contralateral renal I/R, including tubular dilation and necrosis (inflammatory cell infiltration and cellular edema in the tubular interstitium) in the renal cortex and outer medulla. Red Propolis administration 3 days before the procedure and one hour prior to surgical procedure or ischemia attenuated kidney damages and significantly decreased tubular necrosis score [27]. Conversely, in Orsolic et al. study, renal examination revealed *corpuscular changes* (narrowing or reduction of Bowman's space due to the expansion of mesangial and/or endothelial cells of the glomerulus, and the presence of columnar cells in the parietal layer of Bowman's capsule), *tubular alterations* (the presence of necrotic cells, basophilic cells, cytoplasmic vacuolization, vacuole-like spaces in the tubular lumen, epithelial flattening with or without intraluminal eosinophilic mass, and dilated tubules), and *interstitial disorders*, and further impaired function in the kidneys of control diabetic mice. Administration of 50 mg/kg/day of Propolis [EEP or water-soluble derivative of Propolis (WSDP)] for seven days did not improve renal histopathology in diabetic mice. However, there were fewer basophilic and more dilated tubules in the EEP group, and more extensive lymphocyte infiltrations, as well as more dilated tubules in the outer cortex in the WSDP group, compared to control mice [28].

### Propolis and renal function in kidney disease

#### Animal studies

Among the fourteen selected animal studies, six had evaluated the impact of Propolis on renal function. Rabey et al. showed that 20% w/w Propolis methanol extract supplementation for 4 weeks significantly decreased urinary albumin excretion (UAE) in diabetic rats [24]. Also, in a study by Abo-Salem et al., 100, 200, 300 mg/kg/day ethanol extract of green Propolis administration for 40 days significantly decreased UAE in all dosages of supplementation in diabetic rats [31]. In two other studies, administering 100 mg/kg/day ethanol extracted Chinese or Brazilian Propolis for 8 weeks in diabetic rats could decrease urinary albumin excretion rate (UAER) in both groups but had no significant effects on creatinine clearance rate (CCR) [29, 30]. In another study by da Costa et al., involving rats exposed to unilateral nephrectomy and contralateral renal I/R, administration of 150 mg/kg/day of RP 3 days before the procedure and one hour prior to surgical procedure or ischemia caused a significant increase in creatinine clearance (ClCr) [27]. Also, Teles et al. reported that in 5/6 renal removed rats, administration of 150 mg/kg/day alcoholic extract of RP for 2 months led to a significant decrease in proteinuria [10].

#### Human studies

All of the human studies included in this article have assessed the effects of Propolis on renal function. In the study by Fukuda et al., Propolis supplementation with a 226.8 mg/day dose for 8 weeks in patients with type 2 diabetes did not change estimated GFR (eGFR) and urinary albumin-to-creatinine ratio (UACR) significantly [33]. Also, Zakerkish et al. reported that 1000 mg/day of Propolis supplementation for 90 days in T2DM patients had no significant effects on eGFR [32]. Silveira et al. showed that 500 mg/day Propolis supplementation for 1 year in CKD patients, although had no significant effects on eGFR and UACR, could remarkably decrease proteinuria [13].

### Propolis and renal function indicators in kidney disease

#### Animal studies

From the fourteen animal articles reviewed, eleven studies evaluated the effects of Propolis on renal function indicators. In an experimental study by Laaroussi et al. on diabetic rats, administering 100 or 200 mg/kg/day Moroccan Propolis for 16 weeks significantly decreased SCr, urea, and uric acid, and increased total serum protein and albumin; however, serum level of electrolytes including Sodium (Na^+^), Potassium (K^+^), and Chloride (Cl^−^) did not show any significant changes [19]. In another study conducted by El Menyiy et al., administering 50 or 100 mg/kg/day hydroalcoholic extract of Moroccan Propolis for 15 days to diabetic rats caused a significant decrease in urea (only at a dose of 100 mg/kg/day) and SCr, and increase in serum albumin concentrations. The levels of serum protein did not change significantly [21]. Two studies reported the effects of 200 mg/kg/day Propolis intake on CKD experimental models. In Salmas et al. study, Propolis intake for 2 weeks resulted in a significant decrease in renal asymmetric dimethylarginine (ADMA) levels, a NO synthase inhibitor, in hypertensive rats [23]. In another study on diabetic rats by Jabir et al., EEP administration for 3 weeks decreased the serum uric acid levels and increased the total serum protein concentrations, both significantly [26]. Similarly, Rabey et al. reported that administration of methanol extract of Propolis (20% w/w) for 4 weeks significantly decreased the serum levels of urea, creatinine (Cr), and uric acid, and increased the urinary Cr and the serum electrolytes levels (restoration of Na^+^ and K^+^ to normal levels) in diabetic rats [24]. Moreover, the effectiveness of 150 mg/kg/day of RP was examined in two articles. In Teles et al. study, a significant decrease in SCr levels and systemic blood pressure (BP) in rats with renal ablation was reported following alcoholic extracted RP intake for 2 months. However, no difference was noted for the levels of renal interstitial cells positive to angiotensin II (AII^+^ cells), which are involved in the HTN development [10]. Moreover, in da Costa et al. study, a statistically significant decrease in serum levels of urea, Cr, and absolute excretion of Na^+^ and K^+^ (as markers of functional tubular viability and renal tubular injury) was noticed following RP pretreatment in rats exposed to unilateral nephrectomy and contralateral renal I/R [27]. Notably, Abo-Salem et al. conducted a study in diabetic rats and observed that an intake of EEP (100, 200, 300 mg/kg/day) for 40 days significantly decreased blood urea nitrogen (BUN) (at the three tested doses) and SCr levels (except at dose of 100 mg/kg/day) [31]. The effectiveness of Propolis types (Chinese or Brazilian) has been examined in two articles. Zhu et al. administered 100 mg/kg/day of EEP to diabetic rats for 8 weeks and reported that only Brazilian Propolis significantly reduced BUN levels, and SCr did not change significantly by both types [29]. In another similar study by Zhu et al., BUN and SCr levels did not change significantly, independent of Propolis types [30]. Effects of Propolis preparations (50 mg/kg/day of ethanolic or aqueous extract of Propolis for 7 days) have been evaluated in Orsolic et al. study. It was shown that serum urea did not change by both preparation methods of Propolis in diabetic mice [28].

#### Human studies

All three randomized clinical trials examined the impact of Propolis on renal function indicators. Silveira et al. reported that BP did not change significantly in CKD subjects supplemented with 500 mg/day Brazilian green Propolis for 12 months [13]. Zakerkish et al. conducted an RCT on diabetic patients and revealed that Propolis supplementation of 1000 mg/day for 90 days did not significantly affect BUN, SCr, and serum uric acid levels [32]. Similarly, in another study by Fukuda et al., it was shown that the administration of 226.8 mg/day Brazilian green Propolis in diabetic subjects for 8 weeks had no effect on the levels of serum uric acid and urine pH [33].

## Discussion

**Fig. 2:**
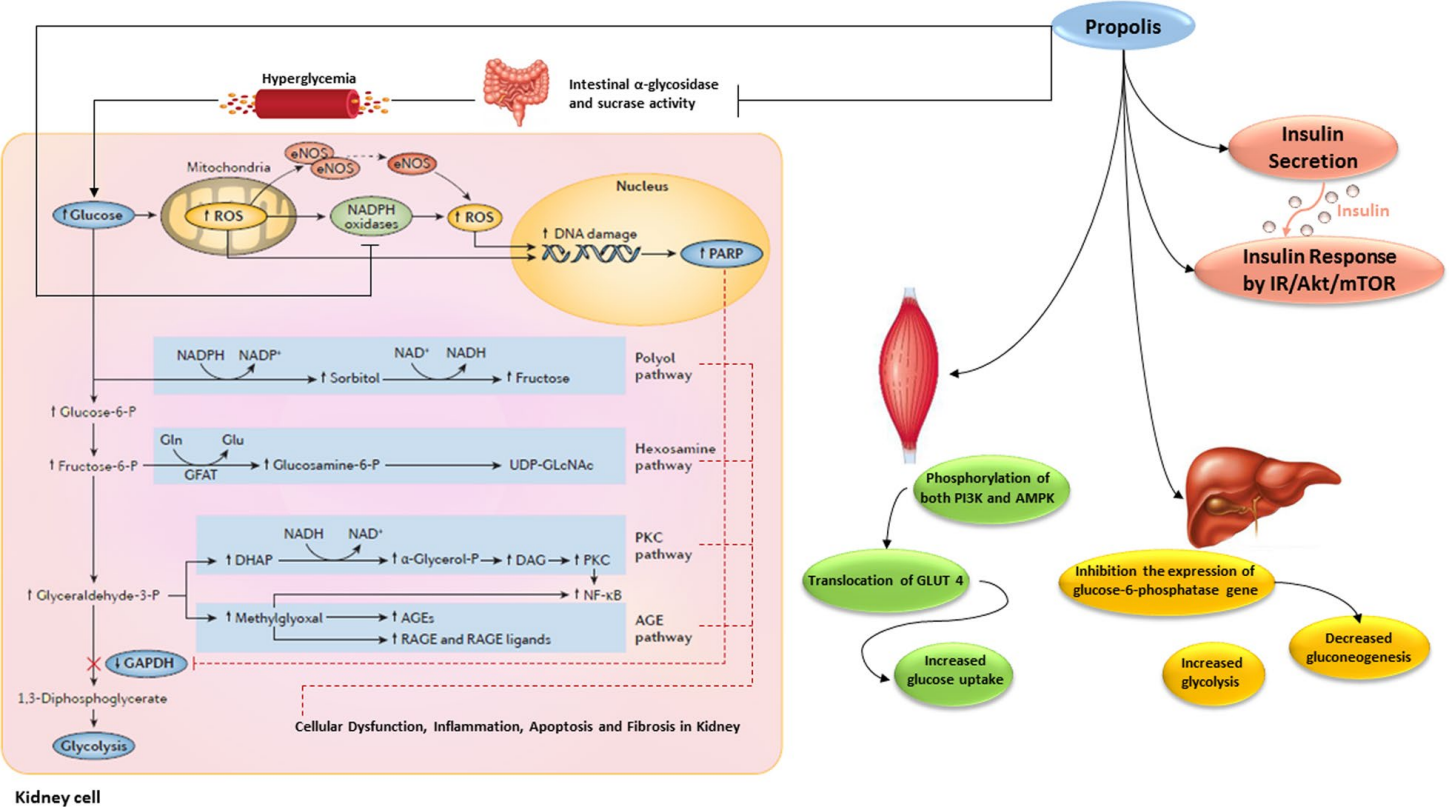
The possible mechanisms for the effects of Propolis on hyperglycemia and hyperglycemia-induced renal damage. In diabetic patients, renal glucose uptake is increased in both the post-absorptive and postprandial states; however, muscle glucose uptake is either normal or reduced. Compensated increased glucose uptake in the kidney enhances ROS generation, eventually contributing to OS and pathogenetic pathways, which lead to renal tissue dysfunction. Propolis, by decreasing intestinal absorption of carbohydrate and expression of gluconeogenic genes in hepatocellular cells and elevating insulin production, cellular sensitivity to insulin, and the level of glycolysis in the liver, could alleviate hyperglycemia and prevent hyperglycemia-induced renal damage. Also, by its antioxidant properties, Propolis can reduce cellular dysfunction, inflammation, apoptosis, and fibrosis in kidney (Figure adapted from Fig. 2. in Ref. (6)). Abbreviations: ROS, reactive oxygen species; eNOS, endothelial nitric oxide synthase; PARP, poly ADP ribose polymerase; GAPDH, glyceraldehyde-3-dehydrogenase; AGE, advanced glycation end-product; DAG, diacylglycerol; DHAP, dihydroxyacetone phosphate; GFAT, glutamine fructose-6-phosphate amidotransferase; NF-κB, nuclear factor kappa B; PKC, protein kinase C; RAGE, receptor for AGE; UDP-GLcNAc, uridine diphosphate N-acetylglucosamine; PI3K, phosphatidylinositol 3-kinase; AMPK, 5'-adenosine monophosphate-activated protein kinase; GLUT 4, insulin-sensitive glucose transporter 4; IR, insulin receptor; Akt, serine/threonine protein kinase B; mTOR, mammalian target of rapamycin

In this systematic review, the effects of Propolis, as a nutrient substance with antioxidant and anti-inflammatory properties, on clinical course of kidney disease and the associated markers were evaluated. The previous systematic review suggests that Propolis may be beneficial for glycemic control in adults with T2DM [56]. The current study showed that Propolis supplementation had a potential effect on improving 2hpp Glc [32] and the percentage of CML [24] in kidney disease. The studies regarding HOMA-β [19, 32] and QUICKI [19] were not enough to make a judgment. On the other hand, eight studies showed a significant reduction of FBS following Propolis supplementation [19–21, 24–26, 30, 31], although no considerable effects were reported in the other three studies [28, 32, 33]. Moreover, despite the significant decrease of HbA1c in three studies [29, 30, 32], two trials showed insignificant reductions [13, 33]. However, the results regarding insulin levels and HOMA-IR were controversial; in three studies [19, 32, 33], the levels decreased, but only in two of them these alterations were significant, suggesting improving the insulin sensitivity [19, 32]. These differences could be explained by the changes in dose and duration of intervention, type and geographical origin of Propolis, and the season in which it was obtained; and also, preparation of Propolis extract with water or ethanol may vary the Propolis main components. Notably, these discrepancies may also be due to variations in sex, age, genetic, physical activity, nutritional intake, gut microbiota, and other confounders, such as the family history of diseases in clinical trial [57]. As a matter of fact, animal studies could be controlled better than human ones in terms of confounders. Therefore, well-designed clinical trials are needed to compare the significant effects of different types of Propolis. The possible mechanisms underlying the glycemic control achieved by Propolis supplementation could be attributed to the existing bioactive compounds, which could increase insulin production or/and cellular sensitivity to it [58]. In a study by Zhang et al. [59], Propolis extract compared to synthetic α-glycosidase inhibitor such as acarbose showed more potent inhibitory effects on α-glycosidase and intestinal sucrase. Also, Matsui et al. [60] pointed that the anti-hyperglycemic effect of Propolis comes from the inhibition of glucose production from dietary carbohydrates and highly suggested this resinous substance for controlling or delaying the postprandial glucose elevation and improving insulin resistance as well. Furthermore, Propolis extract not only reduces the intestinal absorption of carbohydrates, triggers glucose uptake and the translocation of insulin-sensitive glucose transporter (GLUT) 4 in peripheral tissue like skeletal muscle cells by inducing phosphorylation of both phosphatidylinositol 3-kinase (PI3K) and 5'-adenosine monophosphate-activated protein kinase (AMPK) [32, 61]. Of note, Propolis may suppress the gluconeogenic genes in hepatocellular cells, especially the glucose‐6‐phosphatase coding gene [62]. Increasing glycolysis and glucose utilization in the liver has been suggested as another route for Propolis’s mechanism of action [32]. Chronic hyperglycemia is the main cause of micro-and macro-vascular complications of diabetes and the leading reason for CKD [63, 64]. Most previous studies have found that renal glucose uptake in diabetic patients was increased in both post-absorptive and postprandial states; however, muscle glucose uptake was either normal or reduced [65, 66]. Compensated increased glucose uptake in the kidney not merely due to the mass action effects of hyperglycemia but because of increased renal glucose fractional extraction by overexpression of GLUT-1 increases the generation of reactive oxygen species (ROS) and activates mediators of intrarenal inflammation. It also suppresses intracellular antioxidant defense mechanisms, eventually contributing to OS, and leading to renal tissue dysfunction [63, 64, 66–68]. Oxidative stress, as one of the major factors of DN, may activate NF-κB, which controls the expression of a cascade of pro-inflammatory molecules contributing to the progression of apoptosis and renal dysfunction [57, 68]. Also, increased formation of AGEs caused by persistent hyperglycemia induces the AGE-RAGE (advanced glycation end products-receptor for advanced glycation end products) interaction in the kidney, which contributes to the activation of intracellular ROS generation [63]. Briefly, stimulating the ROS-mediated pathways such as NF-κB, protein kinase C (PKC), angiotensin II synthesis, polyol pathway flux, hexosamine pathway flux, and AGE formation due to the hyperglycemia leads to renal lesions eventually [30]. Besides, it has been assumed that chronic hyperglycemia induces hemodynamic changes such as elevated mechanical tension and frictional forces to the glomeruli, and it contributes to renal injury by increased secretion of many pro-inflammatory cytokines and growth factors with further stimulation of the OS [63]. Therefore, Propolis's potential for glycemic control can help to prevent the initiation and progression of kidney disease (Fig. 2).

**Fig. 3:**
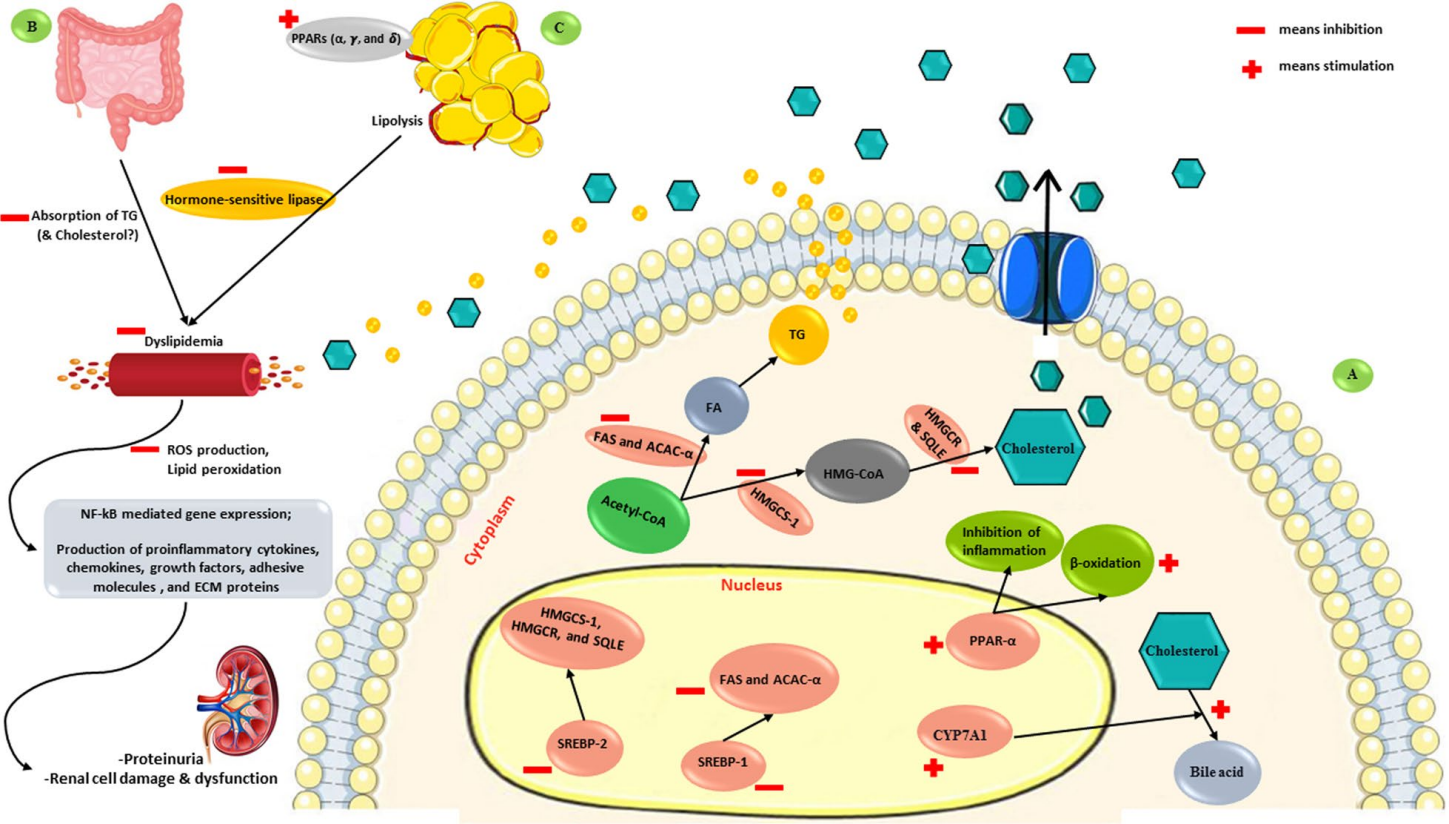
The important suggested mechanisms for the effect of Propolis on dyslipidemia in **A** liver by inhibiting cholesterol and triglyceride synthesis and inducing ß-oxidation and cholesterol-bile acid turnover, **B** gastrointestinal system by inhibiting the absorption of triglyceride and probably cholesterol, and **C** adipose tissue by regulation of fat accumulation and lipolysis and dyslipidemia-induced renal damage. Abbreviations: CYP7A1, Cholesterol 7α-hydroxylase; SREBP, Sterol regulatory element-binding proteins; FAS, fatty acid synthase; ACAC-α, acetyl-CoA carboxylase α; HMGCS-1, 3-hydroxy-3-methylglutaryl-Coenzyme A synthase 1; HMGCR, 3-hydroxy-3-methylglutaryl-Coenzyme A reductase; SQLE, Squalene Epoxidase; PPAR, peroxisome proliferator-activated receptor; FA, fatty acid; TG, triglyceride; ROS, reactive oxygen species; NF-κB, nuclear factor kappa B; ECM, extracellular matrix

Dyslipidemia is one of the common features of kidney disease and a modifiable risk factor for CVD, the leading cause of mortality in AKI and CKD [6, 12, 69–71]. According to the studies reviewed here, five animal studies and two human ones assessed lipid profile in DN. The results on TC levels were controversial, with decreased levels in four studies [19, 21, 29, 31] and no significant changes in three studies [28, 32, 33]. Similarly, evidence regarding HDL-C levels was inconsistent; while the results of the four studies showed considerably increased levels of HDL-C [19, 21, 31, 32], there was not any significant increase in the other two [29, 33]. Regarding TG levels, the results were controversial. Although there were decreased TG levels in all seven studies [19, 21, 28, 29, 31–33], significant changes were shown only in three articles [19, 21, 31]. Markedly, Propolis supplementation did not improve LDL-C concentrations, as it decreased in three studies [19, 21, 31] and did not show significant changes in the other three ones [29, 32, 33]. Moreover, the findings revealed that the effects of Propolis on VLDL-C were inconsistent [19, 21, 32]. The number of articles reporting RLP-C was not sufficient to make a judgment [33]. Dyslipidemia can stimulate OS and inflammation in the body, leading to vascular and renal injury [29, 32, 72]. On the other hand, kidney disease, regardless of the underlying cause, can result in dyslipidemia phenotype, which occurs in CKD patients, with this frequently observed pattern: increased TG, VLDL-C, intermediate-density lipoprotein (IDL), chylomicron remnants, and oxidized lipoproteins, decreased HDL-C, and various levels of serum TC and LDL-C concentrations [12, 71]. According to previous studies, Propolis may prevent or attenuate dyslipidemia by improving the glycemic status and relieving the OS [73–75]. In general, the possible lipid-lowering effects of Propolis are mediated by regulation of the lipid absorption, metabolism, accumulation, excretion, and synthesis in the body [74–76]. It is proposed that Propolis acts through up-regulation of the PPAR-γ in the adipose tissue, which is a therapeutic target in DM, metabolic syndrome, and CVD, and involved in improving insulin sensitivity, inflammation, and dyslipidemia [57, 76, 77]. Moreover, Propolis can up-regulate the PPAR-α and PPAR-δ, that control genes involved in lipid catabolism and free-fatty acid β-oxidation, as well as down-regulate sterol regulatory element-binding protein-1 (SREBP-1) and consequently fatty acid synthase (FAS) and acetyl-CoA carboxylase α (ACAC-α), leading to decreased fatty acid synthesis in the liver [57, 74, 76]. Also, Propolis administration results in down-regulation of SREBP-2, 3-hydroxy-3-methylglutaryl-Coenzyme A synthase 1 (HMGCS-1), 3-hydroxy-3-methylglutaryl-Coenzyme A reductase (HMGCR), and squalene epoxidase (SQLE), leading to decreased hepatic cholesterol synthesis [57, 73, 74, 76]. Moreover, Propolis improves the lipoprotein lipase activity in the vessels (similar to lipid-lowering medications, such as fibrates) while inhibiting the hormone-sensitive lipase activity in adipose tissue, all leading to improved dyslipidemia [73, 75]. On the other hand, Propolis probably causes increased cholesterol 7α-hydroxylase (CYP7A1) expression and leads to more neutral bile acid biosynthesis from cholesterol [74]. Besides, Propolis promotes protein expression of ATP-binding cassette transporters in the liver, which is related to reverse cholesterol transport and HDL-C formation [32]. In the gastrointestinal system, Propolis possibly inhibits the intestinal absorption of dietary lipids (TG and probably cholesterol) [76]. In addition, overweight/obesity is considered a significant risk factor for the development and severity of decreased GFR, regardless of the metabolic status [78, 79]. As Propolis could regulate the microbiota profile (both composition and function), leptin secretion, PPARs (α, γ, and δ) action, and lipids metabolism (absorption, lipogenesis, and lipolysis), it can inhibit the weight gain and diminish the visceral adipose tissue accumulation [74, 80–83]. For this reason, Propolis can also reduce the risk of CKD due to its anti-obesity properties [74, 80–83] (Fig. 3).

As mentioned earlier, OS is one of the leading risk factors for vascular and renal tissue dysfunction; therefore, the effect of Propolis on OS in kidney disease was evaluated in the current study. In a systematic review by Kocot et al. [15], it has been reported that Propolis as a natural agent can counteract the effects of OS, which is involved in the pathogenesis of various diseases. In this systematic review, Propolis supplementation found to be effective in decreasing the levels of MDA in serum [24, 26, 29–31], renal [22, 24, 25, 27–31], liver [28–30] and urine [27], and urinary levels of reactive oxygen metabolites (TBARS) [10], as lipid peroxidation indicators, significantly. Moreover, it had a potential effect on reducing the levels of renal tissue TOS [23] and OSI [23], and 8-OHdG formation [22], as a well-known biomarker of DNA damage in the renal tissue. Nitric oxide, as a reactive nitrogen species (RNS), is an important signaling biological molecule, and excessive NO can react with superoxide molecules producing strong oxidant peroxynitrite that is involved in the pathophysiology of ischemic AKI and DN [27, 30]. Nitric oxide is synthesized by a family of NO synthases (NOS), including neuronal NOS (nNOS), inducible NOS (iNOS), and endothelial NOS (eNOS). Nitric oxide-mediated effects can be beneficial or detrimental depending on the specific risk factors underlying the disease [84]. A reduction in the activity of eNOS is mainly responsible for the elevation of BP, renal impairment due to endothelial dysfunction, and OS [27, 84]. In contrast, abnormal expression of iNOS is likely to be related to the progression of vascular dysfunction, kidney damage, inflammation, and apoptosis. Inhibition of iNOS activity improves renal I/R damage, while eNOS has protective effects on I/R injury [27]. Endothelium-derived NO has an essential role in regulating angiogenesis and decreasing the interstitial fibrosis in the obstructed kidney. However, in rats with CKD, both eNOS and renal and vascular expression of iNOS decreased [84]. In this systematic review, two studies demonstrated a significant reduction in serum NOS without mentioning the type [29, 30], and one study reported a significant increase in renal eNOS score after Propolis administration [27]. The results for serum NO showed insignificant reductions in two studies [29, 30] and a significant decrease in one study [26] following Propolis supplementation that there was not any clear conclusion about it. The effect of Propolis supplementation on the anti-oxidant biomarkers was also evaluated. The findings of these studies showed that Propolis supplementation had a potential role in increasing the levels of serum SOD [24, 26, 29, 30], serum GST [24, 26], liver GPx [29, 30], and renal GSH [22, 27, 31], CAT [29–31], FRAP [25], PON1 [23], HO-1 score [27], and TAS [23]. However, insufficient but also helpful results for serum CAT [24, 26, 29, 30] and renal [22, 25, 29–31] and liver [29, 30] SOD were observed. Nevertheless, Propolis supplementation had no significant effect on liver CAT [29, 30] and serum GPx [29, 30], and the results regarding renal GPx levels [25, 29, 30] were also controversial. Antioxidant properties of Propolis depend on the ingredients possessing phenolic characteristics such as phenolic acids and flavonoids, mainly due to their structure [15].

Inflammation is a crucial criterion for the development and progression of AKI and CKD [11, 24, 70, 85, 86]. In this systematic review, inflammatory markers were evaluated in kidney disease, showing a significant reduction in the levels of renal NF-κB [23], serum immunoglobulins [24], renal ED-1^+^ cells [10], and urinary MCP-1 [13] following Propolis supplementation; while IL-1β levels did not change significantly [32]. Notably, results on IL-6, TNF-α, and hs-CRP were controversial. Serum and renal IL-6 levels decreased in one animal study after Propolis administration [24]; however, in two trials, serum IL-6 levels did not change significantly [32, 33]. Although serum levels of TNF-α decreased in all three human studies [22, 32, 33], these improvements in only two of them reached the level of significance [22, 32]. Serum concentrations of hs-CRP were also assessed in two human studies; its levels declined in one study [32], with no change in another one [33]. More human studies are needed to determine the exact effects of Propolis on inflammation in patients with kidney disease. Oxidative stress-induced systemic and local renal inflammation develops as a cause and/or consequence of renal injury, hyperglycemia, or dyslipidemia [1, 11, 12, 70, 72, 87, 88]. It is shown that Propolis can potentially work as an anti-inflammatory agent in almost all inflammation stages due to the antioxidant and lipid-lowering effects of its flavonoids, especially CAPE, galangin, artepillin C, and quercetin [14, 42, 57, 89–95]. Propolis exerts its effects through different mechanisms; the most important ones are inhibition of: expression and production of NF-κB and other signaling pathways (such as Toll-like receptor (TLR), JAK-STAT Protein kinase B (Akt), and lipopolysaccharide-induced signaling pathways), expression and activation of pro-inflammatory molecules (chemokines, prostanoids, growth factors, immunoglobulins, and cytokines), and expression and activation of iNOS [14, 23, 90, 91, 93, 96–100]. As mentioned earlier, Propolis can also up-regulate PPAR-α and PPAR-γ; therefore, it may promote PPARs-related anti-inflammatory and renoprotective effects [74, 76, 77, 101].

Results from eight animal studies showed that Propolis supplementation had potential effects on the prevention or restoration of renal tissue damages in kidney disease. The findings of three studies involving non-DM models of nephropathy showed that Propolis causes a lower slope in GS, tubular inflammation, tubular dilation and necrosis, renal congestion, renal hemorrhage, renal hydropic degeneration, and renal fibrosis [10, 22, 27]. Also, according to the results of five studies on DN, Propolis administration resulted in preserving the Bowman's capsules and decreasing vascular congestion, collapsed glomerular tuft, GS, the volume of the mesangial cells, GBM thickness, GA, vacuolization of the renal tubular epithelial cast, tubular dilation and atrophy, and interstitial inflammation and hemorrhage [24–26, 30]. However, in one study using the lowest dose, Propolis did not improve the corpuscular, tubular, and interstitial changes in diabetes models [28]. Moreover, evidence regarding the effects of Propolis on kidney weight was controversial; it decreased in three studies [19, 25, 31] but did not change in one another study [29]. The renal injury occurs because of metabolic and hemodynamic changes, mainly hyperglycemia, OS, inflammation, HTN, and dyslipidemia [6, 8, 10, 31, 57, 85, 102–105]. Regardless of the causes, kidney injury induces a repair process [85], however dysregulation of this process results in a vicious cycle of injury, leading to kidney maladaptation, dysfunction, scarring, and finally CKD [85]. This maladaptation advances the CKD progression in the way of occurring the cell activation, inflammation, fibrosis, tubuloglomerular feedback, and metabolic response (involving glomerular hyperfiltration, increased tubular activity, and hypoxia) [85, 106]. Finally, as a direct consequence of CKD, a whole model of the progressive nephropathies happens: GS, increased production of matrix proteins, proliferation and hypertrophy of mesangial cells, tubulointerstitial proliferation, interstitial infiltration, inflammation and fibrosis, systemic and arterial HTN, impaired renal function, and proteinuria [6, 10, 85, 105, 107]. Besides, it should be noted that kidney weight, as another indicator for kidney morphology, changes in the early stages of kidney disease due to disturbed metabolism and increased tubular activity [25, 31].

The effect of Propolis supplementation on renal function was evaluated in several studies. Obtained results from the current systematic review revealed that Propolis administration might reduce UAE [24, 31], UAER [29, 30], and proteinuria [10, 13] significantly. Moreover, two studies reported remarkable increased serum total Protein [19, 26] and serum albumin [19, 21], which may indicate decreased proteinuria following supplementation with Propolis. Notably, six studies showed a significant reduction in SCr level due to Propolis supplementation [10, 19, 21, 24, 27, 31], while no significant effects were reported in three other studies [29, 30, 32]. Besides, Propolis was found to be able to increase ClCr [27] and urinary Cr [24], but had no significant effects on CCR [29, 30], UACR [13, 33], and urine pH [33]. While Propolis supplementation did not significantly affect eGFR [13, 32, 33], in two clinical trials, it was decreased compared to the baseline in patients who took the placebo and not the Propolis [32, 33]. Results regarding serum levels of uric acid, urea, and BUN were promising so that in three animal studies, serum levels of uric acid were decreased [19, 24, 26], but no significant effects were observed in two clinical trials [32, 33]; despite the significant reduction of serum levels of urea in four studies [19, 21, 24, 27], one trial showed insignificant alterations [28]; in addition, BUN levels were significantly decreased in two of four studies [29, 31]. Results demonstrated a significant reduction in absolute excretion of Na^+^ and K^+^ as the markers of functional tubular viability and renal tubular injury [27] and a significant increase in serum Na^+^ and K levels (restoration of Na^+^ and K^+^ levels to normal) after Propolis administration in one study [24], but no significant changes in another one [19]. The effect of Propolis on BP- as one of the leading causes of kidney disease- was also investigated. One study demonstrated a significant reduction in ADMA levels, which is likely to lead to endothelial dysfunction and increased in patients with HTN [23]. In another study, it was shown that although Propolis administration had no significant effect on AII^+^ cells, it could significantly reduce systemic BP [10]. However, in a clinical trial conducted by Silveira et al. [13], following Propolis supplementation, the mean systolic and diastolic blood pressures remained stable during the follow-up period, without statistically significant between groups.

Taken together, mounting evidence indicated that supplementation with Propolis in kidney disease attenuated hyperglycemia, systemic and renal OS, systemic and glomerular BP, and leading to less kidney damage and proteinuria. In addition, the histological assessment showed that Propolis might be effective in renal lesions. Advanced renal injury may already be present even in newly diagnosed diabetic and non-diabetic nephropathies [108], and renal tissue improvement following Propolis intake may happens even before other metabolic alterations.

The renoprotective effects of Propolis is probably due to the presence of chrysin [93]. From this point of view, the current systematic review results showed that Propolis supplementation had potential effects on the restoration of renal tissue damages and renal function in AKI and CKD with different etiologies. However, the results were not sufficient to determine the effective dosage, duration of supplementation, and type of Propolis.

### Knowledge gaps and future directions

Generally, due to the lack of human trials to understand the exact roles of Propolis in the management of kidney disease, prospective and well-designed studies with larger sample sizes and extended follow-up periods are needed to understand the underlying mechanisms. None of the reviewed studies measured blood concentrations of polyphenolic compounds of Propolis and didn’t assess the effect of the administration route on bioavailability. Future clinical studies should be designed to compare the effect of geographical origins, seasons, and extraction methods.

## Conclusion

Altogether, the current systematic review indicated that Propolis had potential effects on improving AKI and CKD by decreasing FBS, serum, liver, renal, and urine OS, proteinuria, and albuminuria, as well as renal tissue damages. However, the effects of Propolis on HbA1c, insulin, HOMA-IR, lipid profile (TC, TG, HDL-C, LDL-C, and VLDL-C), NO, NOS, serum and renal IL-6, TNF-α, and hs-CRP, SCr, uric acid, urea, eGFR, BUN, BP, and kidney weight in subjects of AKI and CKD were satisfactory. Therefore, studies on the underlying mechanisms of the effectiveness of Propolis supplementation in patients with kidney disease are highly suggested.

## Supplementary Information

**Additional file 1**. PRISMA 2020 Checklist.

## Data Availability

The datasets generated and analyzed during the current study are available from the corresponding author on reasonable request.

## Abbreviations

AKI: Acute kidney injury
CKD: Chronic kidney disease
GFR: Glomerular filtration rate
SCr: Serum creatinine
DM: Diabetes mellitus
HTN: Hypertension
DKD: Diabetic kidney disease
DN: Diabetic nephropathy
ESRD: End-stage renal disease
CVD: Cardiovascular disease
OS: Oxidative stress
PRISMA: Preferred reporting items for systematic reviews and meta-analyses
EEP: Ethanolic extracts of Propolis
HPLC: High-performance liquid chromatography
CAPE: Caffeic acid phenethyl ester
iNOS: Inducible nitric oxide synthase
LD50: Median lethal dose
FBS: Fasting blood sugar
HOMA-IR: Homeostasis model assessment of insulin resistance
HOMA-β: Homeostasis model assessment of β-cell function
QUICKI: Quantitative insulin sensitivity check index
CML: Carboxymethyl lysine
AGEs: Advanced glycation end products
HbA1C: Hemoglobin A1C
2hpp Glc: 2-Hour postprandial glucose
TC: Total cholesterol
TG: Triglyceride
LDL-C: Low-density lipoprotein cholesterol
VLDL-C: Very low-density lipoprotein cholesterol
HDL-C: High-density lipoprotein cholesterol
RLP-C: Remnant-like particle cholesterol
MDA: Malondialdehyde
CAT: Catalase
SOD: Superoxide dismutase
GST: Glutathione-S- transferase
NOS: Nitric oxide synthetase
GPx: Glutathione peroxidase
NO: Nitric oxide
I/R: Ischemic-reperfusion
RP: Red Propolis
GSH: Glutathione
eNOS: Endothelial NOS
HO-1: Heme-oxygenase-1
TOS: Total oxidant status
OSI: Oxidative stress index
TAS: Total antioxidant status
PON1: Paraoxonase
8-OHdG: 8-Hydroxy-2′-deoxyguanosine
FRAP: Ferric-reducing ability of plasma
NF‐κB: Nuclear factor kappa B
TNF-α: Tumor necrosis factor α
IL-6: Interleukin-6
MCP-1: Monocyte chemoattractant protein-1
hs-CRP: High sensitivity C-reactive protein
IL-1β: Interleukin-1β
GS: Glomerulosclerosis
STZ: Streptozotocin
GBM: Glomerular basement membrane
GA: Glomerular area
%GS: Percentage of GS
IG: GS index
WSDP: Water-soluble derivative of Propolis
UAE: Urinary albumin excretion
UAER: Urinary albumin excretion rate
CCR: Creatinine clearance rate
ClCr: Creatinine clearance
eGFR: Estimated GFR
UACR: Urinary albumin-to-creatinine ratio
ADMA: Asymmetric dimethylarginine
Cr: Creatinine
BP: Blood pressure
BUN: Blood urea nitrogen
GLUT: Insulin-sensitive glucose transporter
PI3K: Phosphatidylinositol 3-kinase
AMPK: 5'-Adenosine monophosphate-activated protein kinase
ROS: Reactive oxygen species
PKC: Protein kinase C
IDL: Intermediate-density lipoprotein
SREBP-1: Sterol regulatory element-binding protein-1
FAS: Fatty acid synthase
ACAC-α: Acetyl-CoA carboxylase α
HMGCS-1: 3-Hydroxy-3-methylglutaryl-Coenzyme A synthase 1
HMGCR: 3-Hydroxy-3-methylglutaryl-Coenzyme A reductase
SQLE: Squalene epoxidase
CYP7A1: Cholesterol 7α-hydroxylase
RNS: Reactive nitrogen species
nNOS: Neuronal NOS
TLR: Toll-like receptor
Akt: JAK-STAT protein kinase B
